# Degeneration of retinal ganglion cells in diabetic dogs and mice: Relationship to glycemic control and retinal capillary degeneration

**Published:** 2013-06-27

**Authors:** Scott J. Howell, Mena N. Mekhail, Rami Azem, Nicole L. Ward, Timothy S. Kern

**Affiliations:** 1 Department of Opthamology, Case Western Reserve University, Cleveland, OH; 2 Department of Dermatology and Neurosciences, Case Western Reserve University, Cleveland, OH; 3 Department of Medicine and Pharmacology, School of Medicine, Case Western Reserve University, Cleveland, OH

## Abstract

**Purpose:**

The purpose of this study was to investigate (*i*) the effect of diabetes on retinal ganglion cell death in diabetic dogs and mice, (*ii*) the effect of prolonged glycemic control on diabetes-induced death of retinal ganglion cells, (*iii*) whether retinal ganglion cell death in diabetes is associated with degeneration of retinal capillaries, and (*iv*) the effect of diet on diabetes-induced degeneration of retinal ganglion cells in mice.

**Methods:**

Diabetes was induced in dogs using streptozotocin, and levels of glycemic control (good, moderate, and poor) were maintained for 5 years. Diabetes was studied in two mouse models (diabetes induced in C57Bl/6J mice using streptozotocin and spontaneously diabetic Ins2Akita mice). Retinal ganglion cell death was investigated by counting the number of axons from the ganglion cells in the optic nerve and with terminal transferase deoxyuridine triphosphate nick-end labeling and annexin V staining in mice.

**Results:**

As reported previously, the development and severity of vascular lesions of diabetic retinopathy in diabetic dogs were strongly associated with glycemic control. Loss of retinal ganglion cells was extensive in dogs kept in poor glycemic control, and was essentially prevented in diabetic dogs kept in good glycemic control for the 5 years of study. In contrast, “moderate” glycemic control (intermediate between poor and good glycemic control) caused a significant increase in vascular pathology, but did not cause loss of retinal axons in the optic nerve. Using this validated optic nerve axon counting method, the two mouse models of diabetic retinopathy were studied to assess ganglion cell death. Despite 10 months of diabetes (a duration that has been shown to cause retinal capillary degeneration in both models), neither mouse model showed loss of optic nerve axons (thus suggesting no loss of retinal ganglion cells). Likewise, other parameters of cell death (terminal transferase deoxyuridine triphosphate nick-end labeling and annexin V labeling) did not suggest ganglion cell death in diabetic C57Bl/6J mice, and ganglion cell death was not increased by a different commercial diet.

**Conclusions:**

Retinal ganglion cell death in diabetic dogs is significantly inhibited by good or even moderate glycemic control. The finding that diabetic dogs in moderate glycemic control had appreciable vascular disease without apparent retinal ganglion cell degeneration does not support the postulate that neural degeneration causes the vascular pathology. Studies of diabetic mice in our colony again fail to find evidence of ganglion cell death due to prolonged diabetes in this species.

## Introduction

Diabetes has long been recognized as damaging the retinal vasculature, but involvement of the retinal neurons is now recognized. There is evidence that at least some retinal ganglion cells (RGCs) are lost in diabetic patients (summarized in [1]), and evidence supporting RGC loss during diabetes is strong in diabetic rat models [2-13]. The degeneration of RGCs has been reported to begin early after the onset of diabetes in rats, and since this neurodegeneration apparently precedes degeneration of retinal capillaries in diabetes, neurodegeneration has been postulated to contribute to capillary degeneration [5]. This postulated relationship between the neural and vascular lesions of diabetic retinopathy has not been tested further to date.

Improved glycemic control has been shown to inhibit the development of the vascular abnormalities that are used to characterize retinopathy clinically, and to inhibit progression to vision-threatening retinopathy [14-18]. The effect of improved glycemic control on neurodegeneration in diabetic retinopathy has not been previously assessed.

Whether neurodegeneration occurs in the RGCs of mice is less clear. Degeneration of RGCs has been reported in C57Bl/6 mice with chemically induced diabetes and spontaneous diabetes [19-24], but others have not been able to confirm these findings [9,25-29].

Since each RGC has one axon in the optic nerve, quantitation of the number of axons in the optic nerve has been used to quantitate the number of RGCs in several studies. In samples from dogs diabetic for 5 years, we use this method to assess (*i*) the effect of glycemic control on the degeneration of RGCs and (*ii*) the possible relationship between degenerative lesions in neural and vascular cells in diabetic retinopathy. Additionally, we use the same axon-counting method (as well as other techniques) to further investigate whether diabetes causes RGC loss in mice.

## Methods

### Animals

Experimental animals were treated in accordance with the Association for Research in Vision and Ophthalmology Statement for the Use of Animals in Ophthalmic and Vision Research.

### Dogs

Young adult dogs (1.5–2.5 years) were randomly assigned to be made diabetic with alloxan or to remain as untreated control animals. Diabetes was induced in fasted dogs by an intravenous injection of alloxan monohydrate (50–60 mg/kg), which were randomly assigned to one of three prospectively identified diabetic groups (poor glycemic control, moderate glycemic control, good glycemic control) or to remain as nondiabetic controls. Animals in poor control were given only enough insulin to minimize bodyweight loss but not enough to prevent chronic hyperglycemia, whereas animals assigned to good control were given enough insulin (twice per day) to normalize blood glucose. Levels of hyperglycemia in the moderate glycemic control group intentionally were kept between poor and good glycemic control. These animals were part of studies on diabetic retinopathy previously published in collaboration with R. L. Engerman [15,30,31]. Methods by which different levels of glycemia were achieved and maintained were published in those prior reports, but involved food and insulin (dogs in the good glycemic control group got only the amount of food that nondiabetic dogs ate plus adequate insulin to normalize blood sugar; dogs in the poor glycemic control group got ad libitum food and subnormal amounts of insulin; dogs in the moderate glycemic control group got ad libitum food plus the same amount of insulin that dogs in good glycemic control got). Hemoglobin A_1_c is summarized at the long-term average of glycemic control. Vascular pathology in these animals (reported previously) is reproduced here for comparison with the new data on retinal neurodegeneration. The present report includes four dogs per experimental group, and all dogs were killed (anesthetized with 1 ml/kg body weight intravenous Nembutal followed by removal of the heart) after five years of study.

### Mice

Two rodent models of diabetes were studied (streptozotocin-induced diabetes and spontaneous diabetes in the Ins2Akita mouse). Diabetes was induced in 8-week-old male C57BL/6J mice (strain number 000,664; Jackson Laboratory, Bar Harbor, ME; n=5) with five serial, intraperitoneal injections of streptozotocin (STZ; 55 mg per kg bodyweight, dissolved in citrate buffer). One to two weeks later, diabetes was confirmed by three fasting blood glucose measurements of >275 mg/dL. For the Ins2Akita mice (C57BL/6-Ins2Akita/J (strain number 003,548); Jackson Laboratory), diabetes developed spontaneously in the male mice (n=11) due to a mutated insulin 2 gene (JAX Communication, No. 5, Mar 9, 2000). The STZ model pertains to type 1 diabetes, whereas the Ins2Akita model shows insulin resistance characteristic of type 2 diabetes [32]. Bodyweight was monitored twice per week, and mice were administered insulin if any weight loss was detected. Glycated hemoglobin (GHb) and blood glucose were measured regularly throughout the duration of study. All animals were fed Harlan TekLad diet 7004, except a group of diabetics and their controls that were fed a diet (# 8604) that had been used by other investigators [33] who detected substantial RGC death in diabetic C57Bl/6J mice. Non-diabetic C57BL/6J mice (n=9) served as controls. Some animals were killed (anesthetized with 1 ml/kg body weight intravenous Nembutal followed by removal of the heart) after 10±1 months of diabetes, and others (for diet studies) after 2 months of diabetes.

### Optic nerve isolation and preparation

Once removed, the canine and mouse optic nerves were fixed overnight in an isotonic solution of 20% paraformaldehyde and 2.5% glutaraldehyde. After rinsing, processing continued using 3% potassium ferrocyanide and 2% aqueous osmium tetroxide solution for 2 h. Following dehydration in a graded series of alcohols and propylene oxide, nerves were embedded in EPON. One-micron-thick sections were then cut on an ultramicrotome Ultracut E microtome (Reichert-Jung, Depew, NY) about 4 mm in back of the globe, and stained with 1% phenylenediamine in methanol solution to stain the myelin sheathes of the nerves, allowing for their subsequent visualization. Thin sections were generated for electron microscopic analysis.

### Optical microscopy and image analysis

Slides were viewed on an Olympus BX-60 microscope (Olympus, Tokyo, Japan; using 40–100×NA 0.75 objectives and DIC optics to increase contrast). Calibrated images were collected using a SPOT RT Slider camera (Diagnostic Instruments, Sterling Heights, MI). Approximately 100 images were needed to cover the area of an entire dog optic nerve. Individual images were stitched together in Adobe Photoshop using the automerge function to produce a composite image of the entire optic nerve cross section. This composite image was then analyzed using MetaMorph imaging software (Molecular Devices, Downington, PA). First, the area of the entire optic nerve was calculated. Because of the large number of axons in the dog optic nerve, sampling was used, and 35%–45% of the total number of axons in each nerve were systematically collected and quantitated. For mice, all axons were counted. Axon diameters and density were determined using software-enhanced analysis of the phenylenediamine-stained nerves. The software identified axons, calculated axon areas, and counted the number and density of the axons.

### Electron microscopy and image analysis

Electron micrographs were captured on a JEOL-JEM 1200 TEM (JEOL, Tokyo, Japan) EX transmission electron microscope at 1200×. The resulting negatives were scanned on a Techtronic Flatbed scanner to digitize them. Once digitized, the images were treated similarly to the optical images above.

### Terminal transferase deoxyuridine triphosphate nick-end labeling in vivo

Retinal cross-sections (fixed in 10% buffered formalin, embedded in paraffin, and sectioned) were stained for the terminal transferase deoxyuridine triphosphate nick-end labeling (TUNEL) reaction (In Situ Cell Death Detection kit, fluorescein; Roche, Mannheim, Germany). For each assay, one section was treated with DNase (50 U/100 µl) for 10 min to fragment DNA as a positive control. The number of TUNEL-positive nuclei was counted per retinal section. The number of TUNEL-positive cells in the diabetic groups was compared to that in the age-matched nondiabetic controls. The TUNEL assay was performed on two different occasions (each yielded similar conclusions), and at the posterior retina and mid-retina.

### Annexin-based cell death detection

This technique is based on a method reported previously [34]. Anesthetized mice were injected intravitreally with 1 µl of annexin V conjugated to Alexa fluor 488 in both eyes. One hour after the injection, the mice were euthanized as described above, and eyes were collected for analysis. For a positive control, ocular ischemia (1 h) followed by reperfusion (24 h) was performed to induce apoptosis in the retinal ganglion cells [35]. Retinas were imaged in three-dimensional plots generated using spinning disk confocal microscopy.

### Capillary degeneration

The retinal vasculature from mice diabetic for 10 months was isolated from formalin-fixed tissue using the trypsin digest technique [27,29,36]. Acellular capillaries were quantitated in four to seven field areas in the mid-retina (200X magnification) in a masked manner. Acellular capillaries were identified as capillary-sized vessel tubes having no nuclei anywhere along their length, and were reported per square millimeter of the retinal area.

### Statistical analysis

All results are expressed as the mean±standard deviation (SD). Groups were compared by analysis of variance (ANOVA) followed by Fisher’s post-hoc test. Differences were considered statistically significant at p<0.05.

## Results

### Dogs

**Figure 1:**
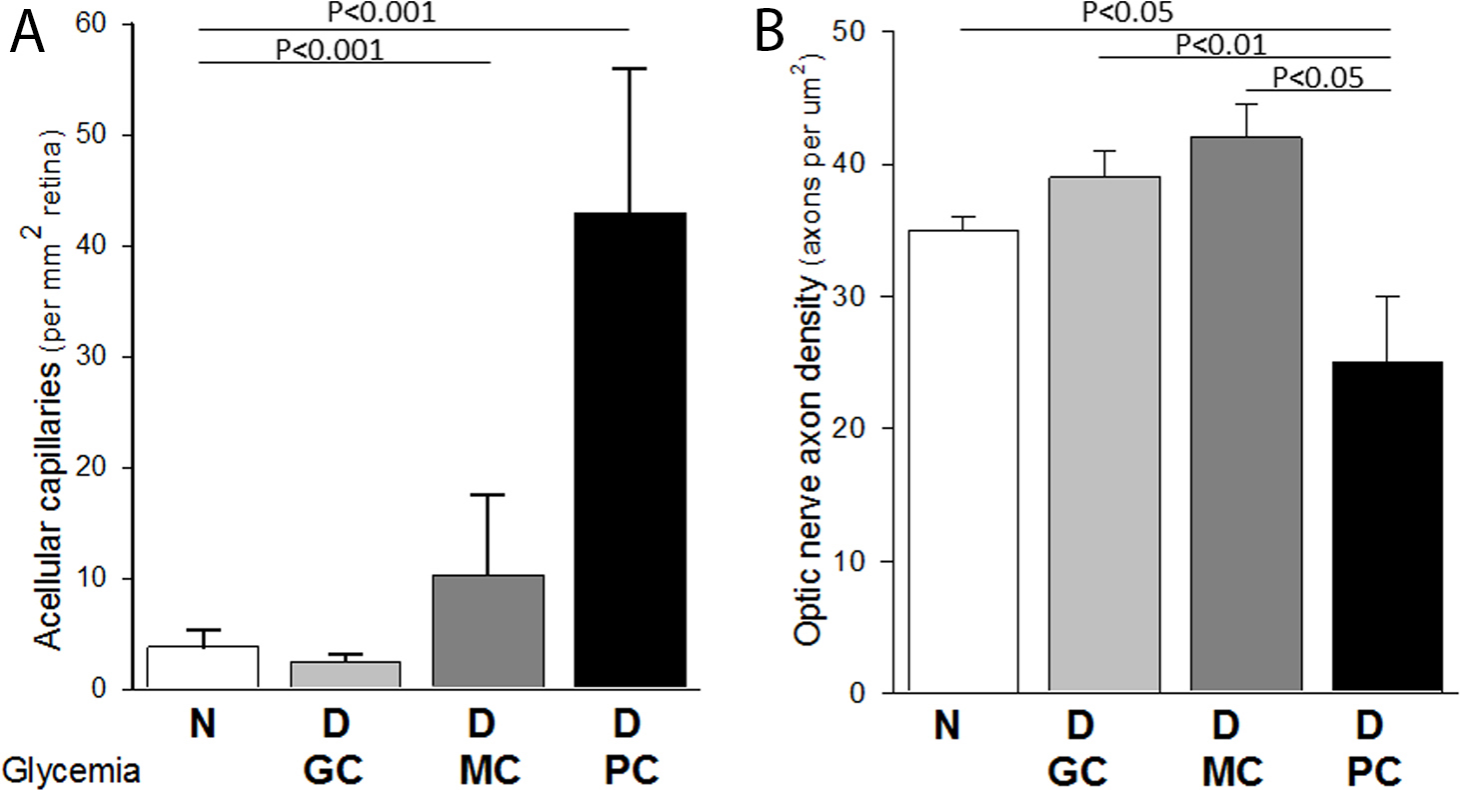
Effect of glycemic control on degeneration of capillaries and retinal ganglion cells in dogs. In dogs diabetic for 5 years, capillary degeneration increased progressively with glycemic control, whereas degeneration of retinal ganglion cell axons in optic nerve did not. **A**: Degenerate (acellular) capillaries increase as glycemic control worsens (data in this table are from animals in previously published studies and have been pooled and reanalyzed [15,30,31]). **B**: In contrast, degeneration of axons in the dog optic nerve does not correlate linearly with glycemic control. Groups are: N, nondiabetic; D GC, diabetic good glycemic control; D MC, diabetic moderate glycemic control; D PC, diabetic poor glycemic control.". n=4 per group. Data is reported as mean±SD.

As reported previously, diabetic dogs were maintained in prospectively assigned levels of glycemic control for 5 years. Capillary histopathology (microaneurysms, capillary degeneration, pericyte loss) was proportional to glycemic control, with animals in poor glycemic control showing appreciably more pathology that the other groups. In contrast, good glycemic control had a clear beneficial effect on the severity of the diabetes-induced retinal vascular disease. Table 1 summarizes the previously published clinical data and parameters of the vascular disease (pericytes ghosts and microaneurysms) in these dogs, and Figure 1 (left) summarizes the capillary degeneration in the same animals. These previously published data on glycemia and vascular pathology have been pooled from several publications [15,30,31], reanalyzed together, and are presented here to allow easy comparison with the data on neurodegeneration.

**Table 1:** Effect of glycemia on microvascular lesions of diabetic retinopathy in dogs diabetic for 5 years^a^

Group	n	HbA1 (%; avg)	Pericyte ghosts	Micro-aneurysms
Nondiabetic	4	5.8±0.2	6.7±2.3	1±1
Diabetic				
Good glycemic control	4	5.6±0.2	3.7±0.1	1±1
Moderate glycemic control	4	7.6±0.1*	8.2±3.3	23±23*
Poor glycemic control	4	9.7±0.6*	39.2±13.2*	72±49*

^a^ Data in this table and Figure 1 is from previously published studies [15,30,31] and has been pooled, recalculated and reformatted. Copyright 1987, 1993, and 2001. From Diabetes^®^, Vol 36, 1987; 898–812, Vol 42, 1993; 820–825, and Vol 50, 2001; 1636–1642. Reprinted with permission of the American Diabetes Association. * Different from nondiabetic at p<0.01

**Figure 2:**
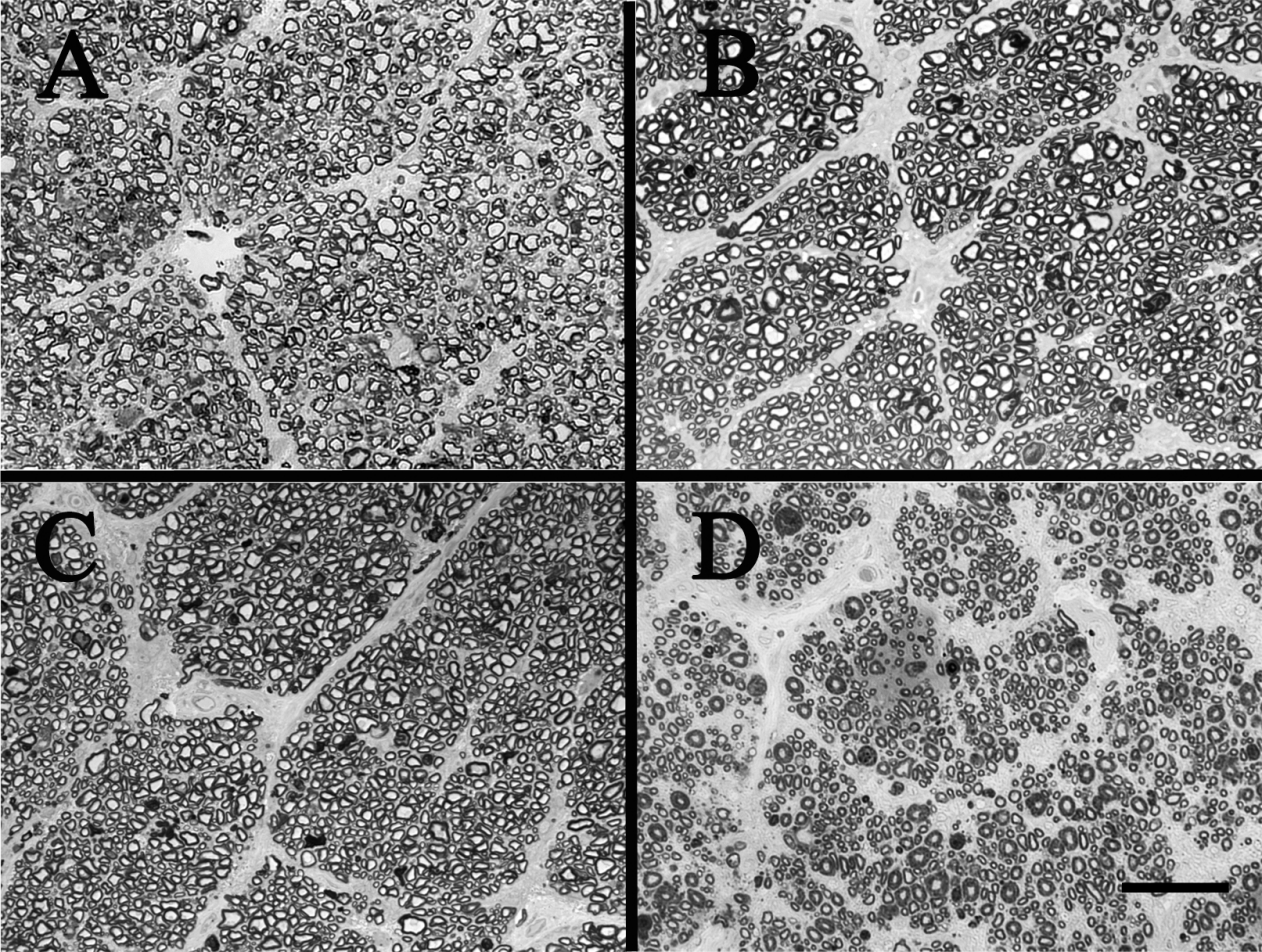
Effect of glycemic control on dog optic nerves as shown with optical microscopy. There is extensive axon loss only in the animals in poor glycemic control for 5 years. Panel **A** shows a representative image from nondiabetic dog. Images **B**, **C** and **D** are from a representative dog in good glycemic control, moderate glycemic control, and poor glycemic control, respectively. Scale bar=50 µm.

The effect of glycemic control on diabetes-induced degeneration of RGCs has not been examined previously. The major parameter examined in this study was mean axon density. Five years of poor glycemic control resulted in a significant loss of axon in the optic nerve (and thus presumably also retinal ganglion cells; p<0.01 compared to nondiabetic) in the diabetic dogs (Table 2 right and Figure 2). The data indicates that 5 years of poor glycemic control resulted in loss of about 40% of all RGCs compared to the age-matched nondiabetic dogs. In contrast, good glycemic control over this 5-year study led to total preservation of the axons (p, not significant compared to nondiabetic animals). Five years of moderate glycemic control, which nevertheless was severe enough to cause a significant increase in vascular pathology (Table 1 and Figure 1), did not cause significant loss of the retinal axons in the optic nerve. Axon density in the moderate glycemic control group was not significantly different from nondiabetic or good glycemic control (p=0.99 and 0.37, respectively), but was significantly different from poor glycemic control (p=0.012). No significant differences were noted in the sizes of individual axons (data not shown).

**Table 2:** Effect of glycemia on optic nerve axons in dogs diabetic for 5 years

Group	n	Axon density (per µm^2^)	Total axons in nerve (x 10^3^)
Nondiabetic	4	0.037±0.002†	116.8±22.4†
Diabetic			
Good glycemic control	4	0.041±0.005†	134.1±28.2*†
Moderate glycemic control	4	0.037±0.008†	121.0±27.3
Poor glycemic control	4	0.026±0.004*	69.1±10.9

* Different from nondiabetic at p<0.01 † Different from diabetics in poor glycemic control at ≤0.01

### Mice

**Figure 3:**
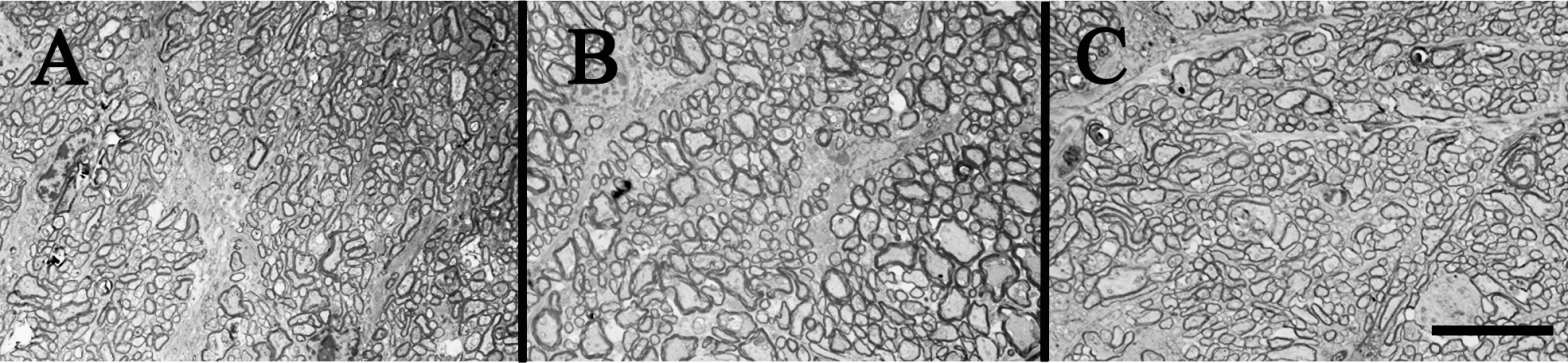
Diabetic and nondiabetic mouse optic nerves. **A**, **B**, and **C** show electron micrographs of diabetic and nondiabetic mouse optic nerves. No differences in axon density were observed between groups. Experimental groups referred to in this figure are: **A**; Nondiabetic (N), **B**; Diabetic-STZ (D STZ), and **C**; Diabetic-Ins2Akita (D Ins2Akita). Scale bar=10 µm.

Using the axon-counting morphometric technique validated in the dog studies, we attempted to determine if optic nerve axons (and ganglion cells) likewise were lost in diabetic mice. Two strains of mice were studied: C57Bl/6J mice in which diabetes had been induced using the B cell toxin, streptozotocin (STZ), and spontaneously diabetic Ins2Akita mice. Both groups of diabetic animals were comparably hyperglycemic (GHb=11.0%±0.6 and 10.9% ±0.6 for STZ diabetes and Ins2Akita, respectively, compared to 3.4%±0.3 for nondiabetic controls). The duration of diabetes in both models was 10 months. Diabetes of 10-month duration did not result in loss of optic nerve axons (as estimated from axon density) in the STZ model (Table 3 and Figure 3) consistent with our previous studies of diabetic C57Bl/6J mice [26-28]. Likewise, direct counting of axons by electron microscopy did not reveal axon loss in C57Bl/6J mice diabetic for 10 months. The size of individual axons was examined, but no significant differences were noted (not shown).

**Table 3:** Mean densities of optic nerve axons in C57Bl/6 mice after 10 mos diabetes.

Group	n	Axon density (per µm^2^)	Total axons in nerve (x10^3^)
Nondiabetic	9	0.212±0.034	21.2±6.0
Diabetic - STZ	5	0.200±0.023	26.2±8.9
Diabetic – Ins2Akita	11	0.255±0.092	23.9±9.8

**Figure 4:**
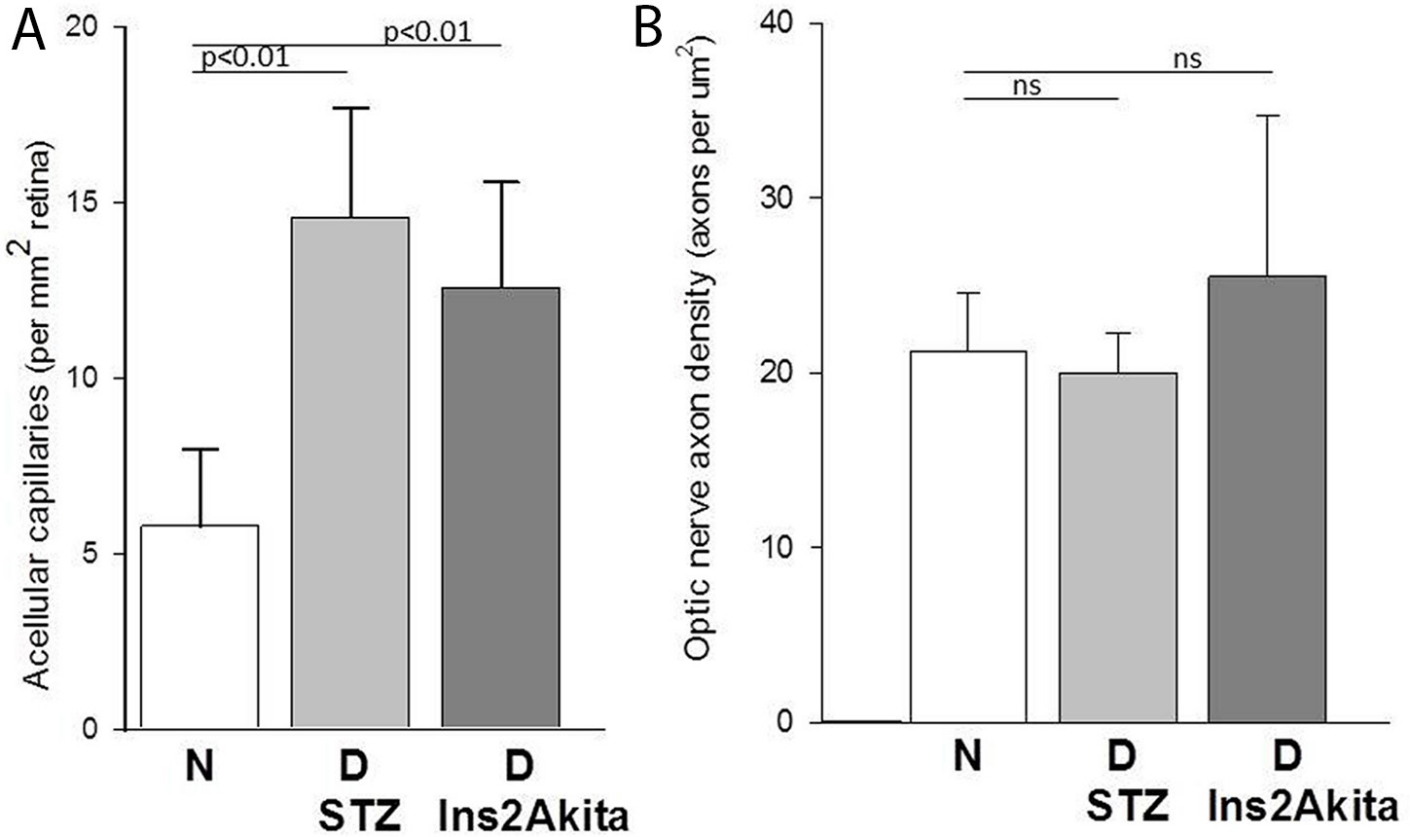
Comparison of the effects of severe diabetes on degeneration of retinal capillaries and retinal ganglion cells in diabetic mice. C57Bl/6J mice diabetic for 10 months show capillary degeneration (**A**) evidenced by significantly increased acellular capillary formation in mice made diabetic with streptozocin (D STZ; n=5) and in spontaneously diabetic Ins2Akita mice (D Ins2Akita; n=11) compared to the nondiabetic controls (N; n=9). No loss of axons from optic nerve was observed between any of the diabetic animals compared to the nondiabetic controls, thus providing no evidence for loss of retinal ganglion cells (**B**).

Since others had reported evidence of diabetes-induced RGC apoptosis in C57Bl/6 mice even at much shorter durations of diabetes [19,23,24], we further assessed RGC death using two additional methods. The first was by injecting fluorescein isothiocyanate–labeled annexin V into the vitreous of nondiabetic and diabetic C57Bl/6J mice [34], and the second was by counting TUNEL-positive cells in retinal cross-sections [26]. Even though both techniques showed that ischemia/reperfusion injury (a positive control) caused RGC death (indicated by the demonstration of numerous positively stained RGCs in mice [not shown]), neither technique demonstrated any differences in the number of apoptotic ganglion cells between long-term diabetic mice and nondiabetic mice. The number of annexin V–positive cells in the RGC layer was 12.2±11.2 and 10.0±5.4 in the diabetic and nondiabetic groups, respectively, and 0.0±0 and 0.1±0.1 TUNEL-positive cells in the RGC layer, respectively (7004 diet). To assess the possible effect of diet on neurodegeneration, we also looked at a different diet with lower levels of antioxidants. Data for mice fed the 8664 diet for the 2-month study likewise showed no increased cell death in the diabetic animals (not shown). This apparent lack of neurodegeneration in both strains of diabetic mice is in contrast to the diabetes-induced significant increase in degenerated capillaries observed in the retinas of the animals (Figure 4).

## Discussion

The clinically demonstrable changes to the retinal vasculature in diabetes have led to the general assumption that retinopathy was solely a microvascular disease. The development of vascular lesions has been found to be closely correlated with glycemic control, with poorer glycemic control greatly increasing the severity and rate of development of the vascular pathology in animals [14,15,17] and in patients [16,18]. The effect of glycemic control on the neural anatomy of the retina in diabetes, however, has not been previously reported. In the present study, the number of axons in the optic nerve was quantitated to assess the effects of diabetes and glycemic control on the number of ganglion cells in the retina (since every ganglion cell has one axon in the optic nerve). These studies showed that the dogs with the poorest glycemia (highest blood glucoses) had the fewest axons in the optic nerve. Conversely, diabetic dogs kept in the best glycemic control were protected from diabetes-induced degeneration of ganglion cell axons. Thus, axon loss in diabetes is not due to “toxic” effects of chemicals to induce diabetes, since axon loss can be inhibited by good glycemic control. Axons in the optic nerve and microvessels respond similarly to glycemia, at least at the two extremes of glycemia.

Dogs in moderate glycemic control developed a severity of vascular pathology that was intermediate between that detected in dogs in good and poor glycemic control. Surprisingly, the moderate glycemic control group did not show axon loss that would indicate neurodegeneration, even though diabetes caused a statistically significant increase in the number of degenerate capillaries in the retina. In fact, the density of axons in the optic nerve of dogs in moderate glycemic control had an axon density similar to that in the nondiabetic animals. Since the dogs in moderate glycemic control developed a statistically significant increase in capillary degeneration without apparent RGC degeneration, this finding does not support the postulate that neural degeneration causes the vascular pathology. The RGCs apparently are more resistant to adverse effects of impaired glycemic control than are cells of the retinal vasculature, at least in moderate levels of hyperglycemia. Whether the vascular and neural lesions of diabetic retinopathy develop by different mechanisms remains to be determined, but the present studies raise that as a possibility.

Based on the success of using the axon-counting technique in the dog model, we employed this technique to revisit the controversy surrounding RGC loss in C57Bl/6J mice. In previous studies of diabetic C57Bl/6J mice, no detectable loss of RGCs was found by investigators previously in retinal cross-sections where the mice were diabetic for up to 1 year [9,25-29], whereas others using the same strain reported that as few as 14 weeks of diabetes was sufficient to detect a significant reduction in the number of cells in the ganglion cell layer compared to that in age-matched nondiabetic mice [19,23,24]. Using optical and electron microscopic methods to analyze mouse optic nerves, we were unable to see any evidence of diabetes causing RGC cell death in the C57Bl/6J mice in the present study. Likewise, diabetic Ins2Akita mice also have been reported to show diabetes-induced degeneration of RGCs [20,21], which we were not able to confirm in the present study of animals diabetic for 10 months. To further investigate our finding that diabetes did not increase RGC death in C57Bl/6J mice, we also employed intravitreal annexin V labeling and TUNEL staining to detect dying cells in the retina. Likewise, neither method showed any evidence of RGC apoptosis in retinas from diabetic C57Bl/6J mice, even though the reaction controls clearly indicated that the techniques were working appropriately. In addition, we considered the possibility that differences in experimental diets fed to the animals might influence diabetes-induced death of retinal ganglion cells. We compared our diet to another diet previously used by others who found diabetes-induced apoptosis of RGCs [33], but found that neither diet increased RGC apoptosis in diabetes, thus indicating that diet is not the cause of the controversy. The difference in diabetes-induced neurodegeneration between laboratories might be explained in part by the recent finding of a mutation related to retinal degeneration (rd8 mutation) in some substrains of C57Bl/6 mice, but not in others [37]. The substrain we used (C57Bl/6J) does not contain the rd8 mutation.

The present results show differences in the effects of poor glycemic control of diabetes on RGC loss between dogs and C57Bl/6J mice. The diabetic mice were at least as hyperglycemic as the diabetic dogs, indicating that the apparent lack of neurodegeneration in the mice was not related to differences in the severity of hyperglycemia. The most obvious difference between the studies of mice and dogs is duration of diabetes. The interaction between the severity and duration of hyperglycemia has been recognized for development of diabetic complications for several decades. Beyond this, however, the information about molecular differences in RGCs between dogs and mice is insufficient to probe this question further at present.

The present studies were limited to neurodegeneration, but diabetes-induced dysfunction of retinal neurons likely precedes degeneration and can contribute greatly to the visual dysfunction that develops in some patients with diabetes. Thus, the focus pertaining to RGCs in diabetes should not be restricted to degeneration.

In conclusion, our data in diabetic dogs demonstrate a beneficial effect of glycemic control on preservation of optic nerve axons (and presumably RGCs), but also show an apparent dissociation between the susceptibility of the retinal vasculature and neurons to degenerate at levels of glycemia between poor glycemia and good glycemic control. It will be informative to determine if the neurovascular relationship is altered regarding function in diabetes. Counting axons in the optic nerve did not indicate a diabetes-induced loss of RGCs in C57Bl/6J mice with chemically induced diabetes or spontaneously diabetic Ins2Akita mice.
